# Nylon 6 Nanofiber Web-Based Signal Transmission Line Treated with PEDOT:PSS and DMSO Treatment

**DOI:** 10.3390/ma14030498

**Published:** 2021-01-21

**Authors:** Sungeun Shin, Eugene Lee, Gilsoo Cho

**Affiliations:** Department of Clothing and Textiles, Yonsei University, Seoul 03722, Korea; seshin@yonsei.ac.kr (S.S.); imujin@yonsei.ac.kr (E.L.)

**Keywords:** Poly(3,4-ethylenedioxythiophene):poly(styrenesulfonate) (PEDOT:PSS), dimethyl sulfoxide (DMSO), Nylon 6 nanofiber web, textile-based signal transmission line, smart clothing

## Abstract

Highly conductive nylon 6 nanofiber webs, incorporating poly(3,4-ethylenedioxythiophene):poly(styrenesulfonate) (PEDOT:PSS) and dimethyl sulfoxide (DMSO), were prepared for textile-based signal transmission lines. To improve the electrical performance of the textiles, they were optimized by the number of coating cycles and the solvent treatment step. The nanofiber web coated four times with PEDOT:PSS showed a six-times reduction in sheet resistance compared to that of once. In addition, the sample treated with both adding and dipping of DMSO showed a significant decrease of 83 times in sheet resistance compared to the sample without treatment of DMSO. Using samples with excellent electrical conductivity, the waveforms of the signal in the time domain were analyzed and shown to have an amplitude and phase almost identical to that of the conventional copper wire. As a result of the S21 characteristic curve, selected textiles were available up to the 15 MHz frequency bandwidth. In the FE-SEM image, it was observed that the surface of the coated sample was generally covered with PEDOT:PSS, which was distinguished from the untreated sample. These results demonstrate that the nanofiber web treated with the optimized conditions of PEDOT:PSS and DMSO can be applied as promising textile-based signal transmission lines for smart clothing.

## 1. Introduction

Biomonitoring smart clothing for healthcare has been developed in response to the emerging focus on the importance of prevention and healthcare [1]. Smart clothing for biomonitoring is applicable in home-training or home-care because it can monitor the wearer’s bioinformation in real-time, regardless of time or space [2].

All smart clothing requires electronic circuits. The signal transmission line is a component of circuits that connect individual electronic components or electronic modules. In smart clothing, textile-based transmission lines are important as power suppliers or signal transmission paths to stably transmit electrical signals [3]. The power supply line must have sufficient electrical conductivity for power transmission efficiency and stability of the supply voltage, and the signal line for high frequency signals needs a wide frequency spectrum [4]. Additionally, the transmission lines should not be distorted in the electric pulse transmitted through the transmission lines, and should minimize the signal loss. Usually, the electrical properties of the transmission lines are evaluated based on the resistance [5] and signal transmission performance such as S-parameter [6]. S-parameters can investigate the power efficiency and bandwidth in the frequency domain. However, S-parameter of the textile transmission lines has not been discussed in the previous literature. In particular, the non-oxidized graphene/PU nanofiber web [7] and the PEDOT:PSS/PU nanofiber web [8] have too high electrical resistance to meet stable power supplies and their frequency characteristics such as S-parameter, and this issue has not been studied. Materials for transmission lines in conventional smart clothing are usually metallic, such as copper, stainless steel, and nickel. Metallic materials are widely used to fabricate transmission lines because they have enough electrical conductivity. For example, textile-based transmission lines have been developed by weaving or knitting with metal yarns, or by printing or depositing using conductive materials on fabric [3,4,5,7,9]. However, metallic materials lack flexibility, which can cause stiffness and discomfort for the wearer [10]. Additionally, metal transmission lines are not durable and are harmful to the skin of the wearer. Therefore, it is necessary to develop a textile-based signal transmission line that is lightweight, flexible, and comfortable to wear.

As an alternative to metallic materials, poly(3,4-ethylenedioxythiophene):poly(styrenesulfonate) (PEDOT:PSS), one of the intrinsically conductive polymers (ICPs), can be considered. PEDOT:PSS is a representative material among thiophene polymers, and is a composite in which positively charged (+) PEDOT and negatively charged (-) PSS are ionically bonded [11]. Its electrical properties can be controlled by adding solvents such as N-methylpyrrolidone (NMP), sorbitol, ethylene glycol (EG), and dimethyl sulfoxide (DMSO) [12,13,14,15]. In addition, PEDOT:PSS is flexible, chemically stable in air, and highly biocompatible, so it can be used in wearable e-textiles or smart clothing [13]. The nanofiber web is a type of nonwoven fabrics, which is consisted of randomly collected textile nanofibers with 1000 nm or less of diameter. It is thin, lightweight, and has excellent air permeability and breathability owing to its micro-porous structure formed between nanofibers [16]. In particular, since the nanofiber web has a large surface area compared to that of conventional textile fibers, the properties or functions can be efficiently maximized when a solution process is applied, and the nanofiber web can be used as e-textiles, smart sensors, and smart clothing [17]. The Nylon 6 nanofiber web has excellent elongation, strength, and abrasion resistance, making it a textile-based transmission line that has durability against the wearer’s body movement when applied to smart clothing.

Previous studies on textile-based transmission lines have mainly focused on the usage of certain temperature or humidity conditions [4,18], or on the structural aspects of transmission lines such as width and arrangement [10,19]. Thus, this study discusses the electrical performance of a textile-based transmission line with enhanced electrical conductivity by DMSO solvent treatment. In particular, DMSO is a nontoxic solvent and improves electrical conductivity with very low toxicity [20,21], and in this study, multi-step DMSO treatment (employed both for pretreatment and post-treatment) was processed to PEDOT:PSS/nylon 6 nanofiber web for improved electrical conductivity. In addition, potential synergies from the use of ICPs and the aforementioned materials may be effective, and so far, the literature has not addressed this issue. Therefore, this study aims to fabricate the textile-based transmission line by using the PEDOT:PSS/nylon 6 nanofiber web and to confirm its highly enhanced electrical conductivity by measuring the electrical resistance. For the performance evaluation, the aim is to analyze the input and output signal waveforms in the time domain, and the power efficiency (S21) in the frequency domain through the S-parameter. The final aim is to verify whether the PEDOT:PSS/nylone 6 nanofiber web-based transmission line properly acts as a transmission line by comparing it to the conventional copper wire.

## 2. Experimental

### 2.1. Materials

Commercially available nylon 6 nanofiber web was purchased (Paradam, Czech Republic) and was used without any treatment. The basic properties are presented in Table 1. Additionally, 1.3 wt% poly(3,4-ethylenedioxythiophene):poly(styrenesulfonate) (PEDOT:PSS) dispersion in aqueous solution was purchased from Sigma-Aldrich, USA, and its specifications are presented in Table 2. For solvent treatment, dimethyl sulfoxide (DMSO) (99.9%) obtained from Deoksan Chemical Industry, Korea, was diluted in distilled water to 5 vol% [22,23]. For enhanced electrical conductivity, 0.05 mL of 5 vol% DMSO was added to 0.5 mL of 1.3 wt% PEDOT:PSS dispersion for the DMSO-treated solution. For post-treatment, a 5 vol% DMSO bath was prepared.

### 2.2. Fabrication of Conductive Textiles

**Figure 1:**
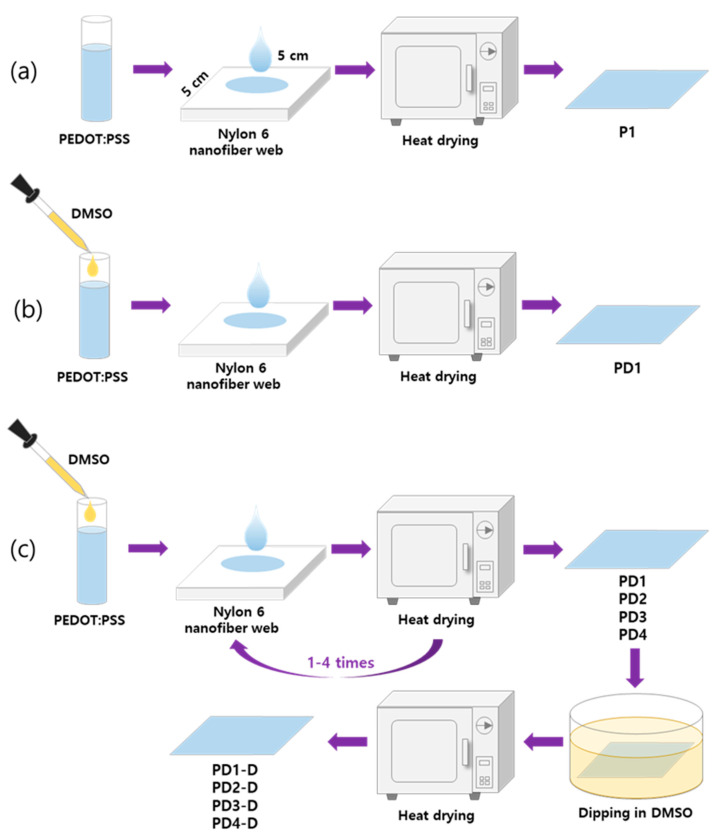
Schematic diagram of the sample fabrication process where (**a**) is treated with pristine PEDOT:PSS only, (**b**) is treated with PEDOT:PSS added with DMSO, (**c**) is repeatedly coated with DMSO-treated PEDOT:PSS and then immersed with DMSO.

As shown in Figure 1, conductive textiles were fabricated. An amount of 0.5 mL of pristine PEDOT:PSS was dropped onto a 5 cm × 5 cm nylon 6 nanofiber web for 15 min at room temperature. Then, the sample was dried at 90 °C for 20 min using a vacuum oven (OV-11, Jeio Tech Co., LTD, Daejeon, Korea) in the atmosphere, and was named P1. As with the previous procedure, the nanofiber web was coated with DMSO-treated PEDOT:PSS. By repeating this process one to four times, samples PD1, PD2, PD3, and PD4 were prepared. After that, the samples coated with the DMSO-treated PEDOT:PSS were dipped in a DMSO bath for 15 min [24] and then dried again in a vacuum oven at 90 °C for 20 min. By this process, samples PD1-D, PD2-D, PD3-D, and PD4-D were prepared. The experimental design for the preparation of samples is shown in Table 3.

### 2.3. Electrical Properties of Coated Nanofiber Web

All samples were kept in ambient atmosphere for 24 h after coating process to ensure stability over time, and then electrical properties were evaluated. To evaluate the electrical properties of the samples, the sheet resistance was measured using a four-point technique (CMT-SR1000N, AIT Co., Ltd., Suwon, Korea) in which four probes were arranged at regular intervals. All data were calculated from the average of the five repeated measurements. To confirm the change in thickness after coating, a cross-sectional image was obtained from FE-SEM (JSM-6701F Plus, JEOL Ltd., Peabody, MA, USA), where the thickness was measured repeatedly three times to calculate the average.

### 2.4. Performance Evaluation of Textile-Based Signal Transmission Lines

**Figure 2:**
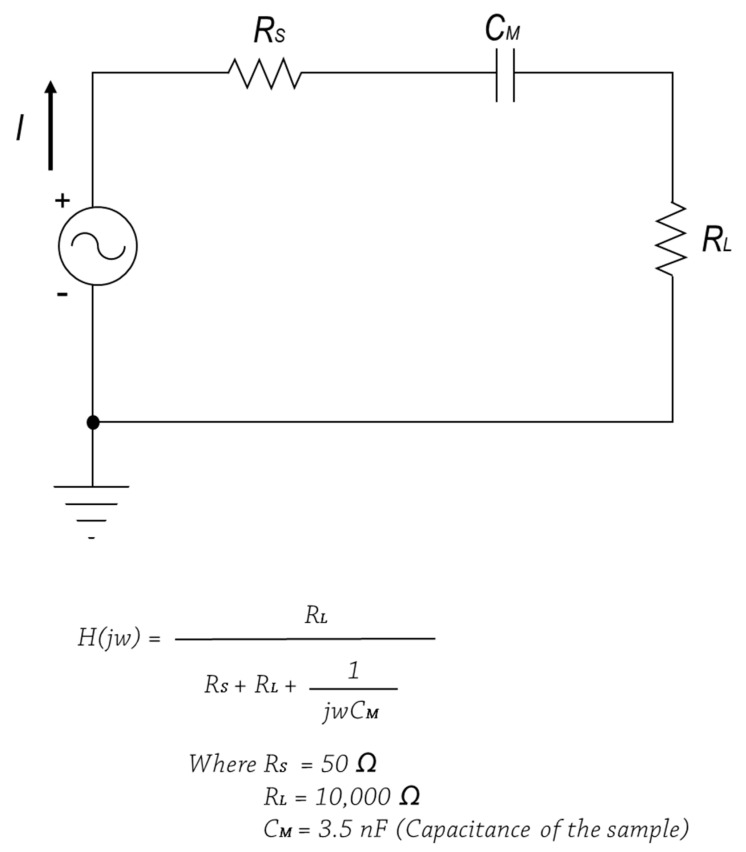
RLC (resistor, inductor, capacitor) circuit for measuring I/O (input and output) signals of the samples.

Based on the results of previous research [7], samples with excellent electrical performance were selected. To confirm whether the selected samples were applicable as textile-based transmission lines, I/O (input and output) signal waveforms were evaluated in the time domain. The RLC (resistor, inductor, capacitor) circuit was prepared as shown in Figure 2. I/O signal waveforms of the samples were measured using an oscilloscope (Wave Surfer 104MXs-B, Teledyne LeCroy, Chestnut Ridge, NY, USA) and compared with the copper wire used as the conventional transmission line. The sample prepared had a size of 4 cm × 2 cm, and input and output terminals were connected to both ends of the samples. All signals were set to 10 Vp-p, 10 kHz of the function generator (Rigol DG4162, RIGOL Techology Co., Ltd., Beijing, China).

Next, the power transmission efficiency of the samples was evaluated by measuring the scattering parameters (S-parameter) in the frequency domain using a vector network analyzer (ZNB8, Rohde & Schwarz, Germany) [5]. Specifically, S21 is the ratio of the input power to the output power expressed in decibel scale as presented below [25,26].

S21 (dB) = 10 × log_10_(P_output, port2_/P_input, port1_) (1)

For this, the samples were prepared in a size of 4 cm × 2 cm, and subminiature version A (SMA) connectors were fixed at both ends of the sample to connect with the coaxial connector port of the vector network analyzer. Calibration was carried out prior to measurement to adjust for instrument error, and the frequency range was set to 100 kHz to 3 GHz. Then, to statistically analyze the I/O signals, the SPSS Statistics Program (ver. 25, IBM, Armonk, NY, USA) was used for all analyses. Linear regression analysis was conducted to investigate the effect of the thickness on the sheet resistance of the samples. Pearson’s correlation analysis was performed to compare the signals between the copper wire and the sample for textile-based transmission lines.

### 2.5. Surface Appearance of the Specimens

The microstructure of the sample surface was examined using field emission scanning electron microscopy (FE-SEM, JSM-6701F Plus, JEOL Ltd., Akishima-Shi, Tokyo, Japan). The surface of the sample prepared by repeated treatment of PEDOT:PSS and DMSO was compared with that of the untreated (UT) nylon 6 nanofiber web sample. All samples were measured at 5000 × magnification.

## 3. Results and Discussion

### 3.1. Electrical Resistance of the Samples

**Figure 3:**
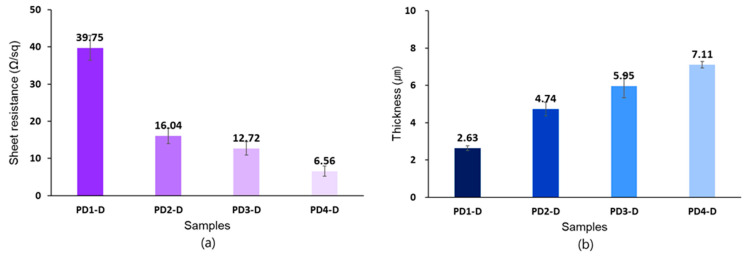
Changes in (**a**) electrical sheet resistance and (**b**) thickness as the coating cycle increased once (PD1-D), two (PD2-D), three (PD3-D) and four times (PD4-D).

Figure 3a presents the sheet resistance of the samples according to the number of PEDOT:PSS coatings. As a result, sample PD1-D had a sheet resistance of 39.75 Ω/sq, and sample PD4-D had 6.56 Ω/sq. The sample coated only once showed the highest resistance while the sample coated four times showed the lowest resistance. This indicated that the electrical resistance decreased as the number of coatings increased. Particularly, the sample PD4-D had the lowest sheet resistance (6.56 Ω/sq), which is much lower electrical resistance even compared to the non-oxidized graphene/PU nanofiber web (29.63 Ω/sq) [7], the PEDOT:PSS/PU nanofiber web (232.9~779.3 Ω/sq) [8], the PEDOT-coated polyamide fiber (approx. 2 kΩ) [27], and the glycerol/PEDOT:PSS-coated polyamide fiber (approx. 740 Ω) [28]. Thus, repetitive coating cycles influenced the improvement of the electrical conductivity [29]. Figure 3b shows the thickness of the samples. The thickness of sample PD1-D, PD2-D, PD3-D, and PD4-D ranged from 2.63 µm to 7.11 µm. Thus, the thickness increased as the number of PEDOT:PSS coatings increased, indicating that the amount of conductive material remaining on the surfaces of the samples increased as well. This proves that the contact of the conductive PEDOT domains within the coating layer is increased, thereby enhancing the transport of charge carriers [30].

**Figure 4:**
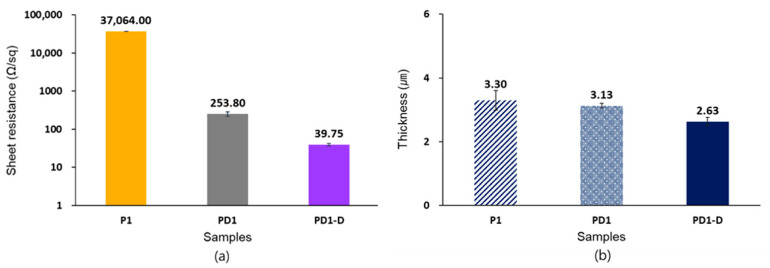
Changes in (**a**) electrical sheet resistance in the logarithmic scale and (**b**) thickness of sample P1, PD1, and PD1-D as the solvent treatment step.

In comparison to the samples coated once, as in Figure 4, the sheet resistance of sample P1, PD1, and PD1-D was 37,064 Ω/sq, 253.8 Ω/sq, and 37.95 Ω/sq, respectively. This indicated that the electrical conductivity improved because there could be possible surface interaction happening between PSS and nylon fiber surface [31]. Additionally, the sheet resistance rapidly decreased, while simultaneously, the thickness decreased [32]. This is because the insulating PSS was removed from the surface of the samples during the DMSO treatment process and the conducting PEDOT particles were connected with each other [24,33]. Thus, the samples displayed enhanced electrical conductivity and sample thickness decreased after DMSO treatment.

On the other hand, the change tendency of the sheet resistance of sample PD1-D, PD2-D, PD3-D and PD4-D was not linear. To investigate the effect of the thickness (X) on the sheet resistance (Y) of the samples, linear regression analysis was analyzed and the results are presented in Table 4. In the case of the samples PD1, PD2, PD3, and PD4, R^2^ of the regression equation was 0.981, however, in the case of the samples PD1-D, PD2-D, PD3-D, and PD4-D, R^2^ of the regression equation was 0.931. This explains that the influence of the thickness on the sheet resistance of the sample PD1-D, PD2-D, PD3-D, and PD4-D was smaller than that of the sample PD1, PD2, PD3, and PD4. In other words, in spite of DMSO post-treatment, the sheet resistance of the samples became less dependent on the thickness change because of a combination of the coating cycles and DMSO post-treatment.

### 3.2. Signal Waveforms of the Samples

Based on the aforementioned results of the electrical properties, sample PD2-D, PD3-D, and PD4-D, which possessed lower resistance, were selected. Using the selected samples, performance was evaluated and compared between the samples and copper wire in the time domain to verify whether the samples are viable for application in signal transmission lines.

**Figure 5:**
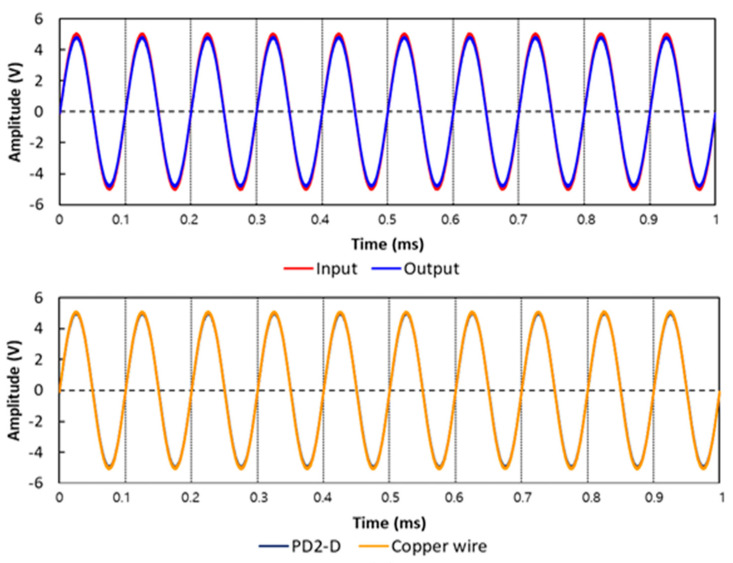
Comparison of waveforms between the input signal and output signal of the samples, and comparison of waveforms between copper wire and the sample PD2-D.

**Figure A1:**
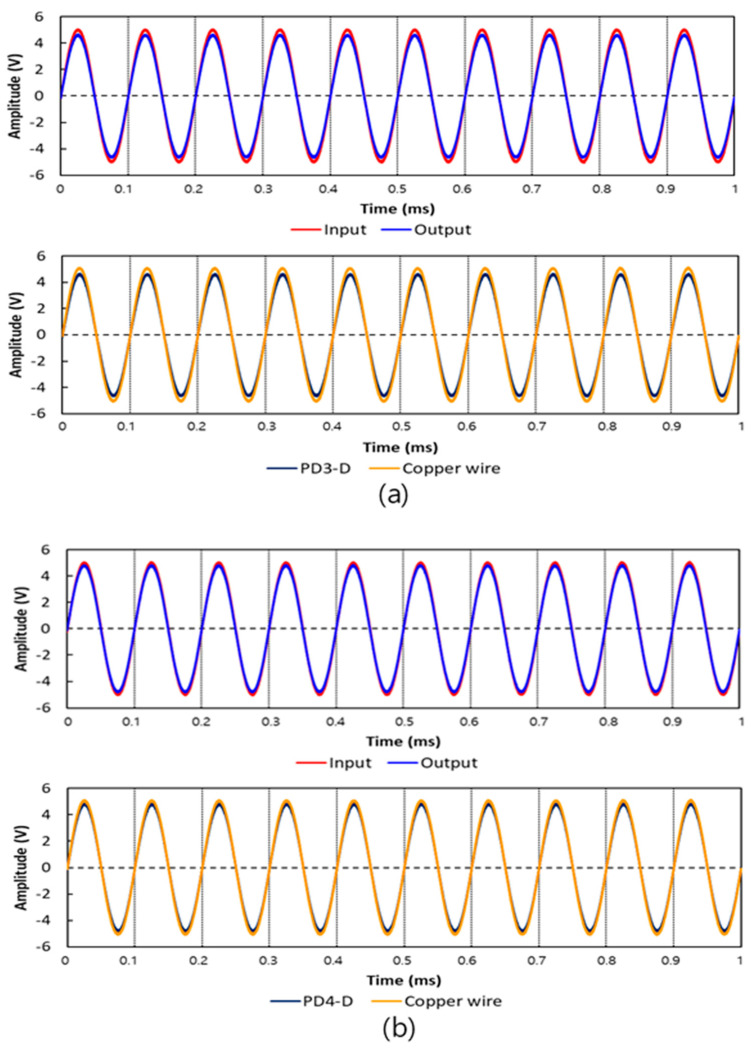
Comparison of waveforms between the input signal and output signal of the samples, and comparison of waveforms between copper wire and the sample (**a**) PD3-D, (**b**) PD4-D.

The waveforms of the input and output signals of the sample PD2-D and copper wire are presented in Figure 5. Sample PD2-D showed that the amplitude and phase of the waveform for the input signal were very similar to those for the output signal. In particular, the output signal of the sample had almost the same waveform as the copper wire. Sample PD3-D (Figure A1a) and PD4-D (Figure A1b) also had signal waveforms similar to those of the copper wire (in Appendix A). In addition, as a result of statistical analysis (Table 5 and Table 6), sample PD2-D had not only a positive correlation between its I/O signals (correlation coefficient of 1.000), but also showed a positive correlation with copper wire (correlation coefficient of 0.999), significantly (*p* < 0.01). In the case of sample PD3-D and PD4-D, the correlation between each signal waveform was proven to be significant (*p* < 0.01). Thus, it was confirmed that the PEDOT:PSS/nylon 6 nanofiber web can be utilized as a textile-based signal transmission line.

### 3.3. S-Parameter of the Samples

**Figure 6:**
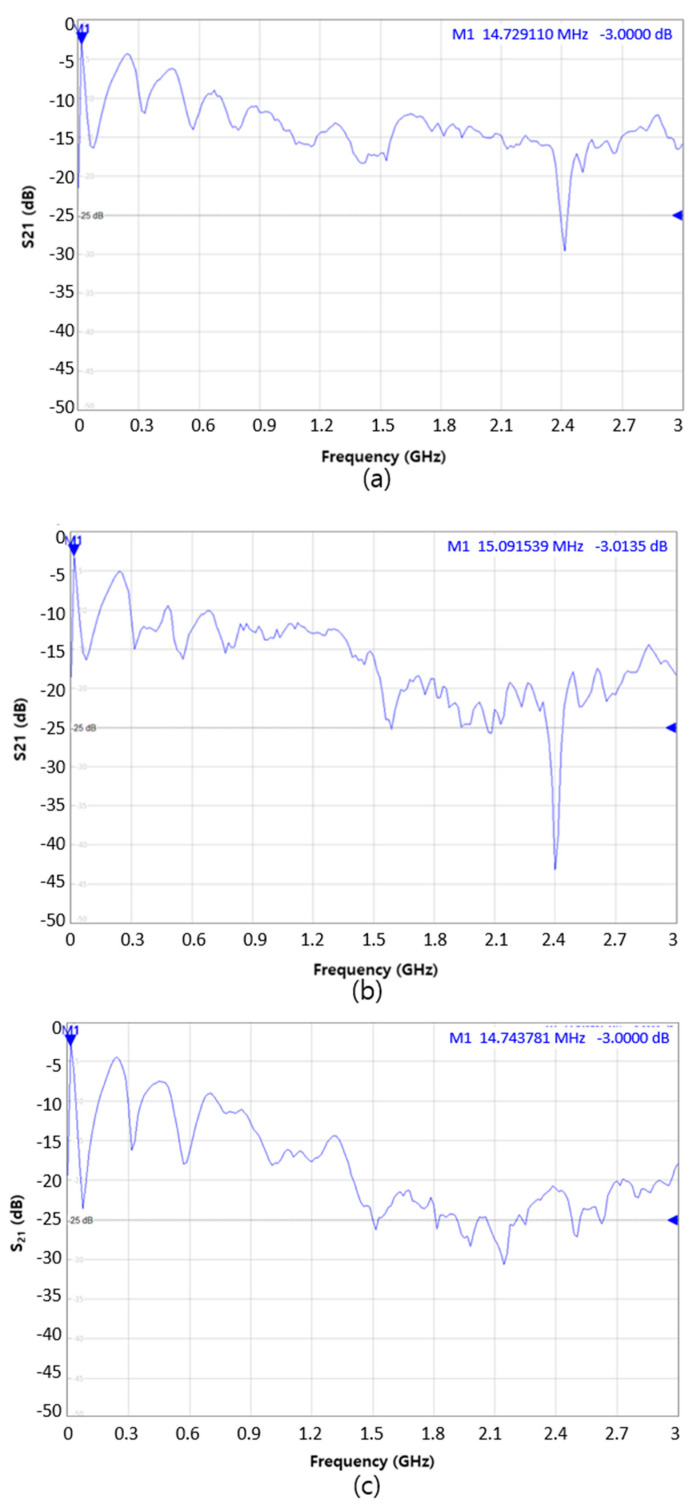
S21 characteristic curves of the sample: (**a**) PD2-D, (**b**) PD3-D, (**c**) PD4-D.

The S21 characteristic curves of the samples are presented in Figure 6. The frequency corresponding to −3 dB is the half point where the output power compared to the input is marked on the graph of each sample and is summarized in Table 7. As a result, the S21 curves of sample PD2-D, PD3-D, and PD4-D showed values below 0 dB and decreased with increasing frequency. This means that the ratio of the output power to the input power decreased more significantly as the frequency was higher. The S21 characteristic curve of sample PD2-D had 14.73 MHz at −3 dB, and those of sample PD3-D and PD4-D had 15.09 MHz and 14.74 MHz, respectively. Therefore, the samples can be used as textile-based transmission lines up to approximately 15 MHz frequency bandwidth. In particular, in this study, we found that there was no difference in the frequency band even though the transmission line had higher electrical conductivity. This implies that the electrical conductivity no longer determines the high-frequency characteristics when it reaches a certain level or higher [9].

### 3.4. Surface Properties of the Samples

**Figure 7:**
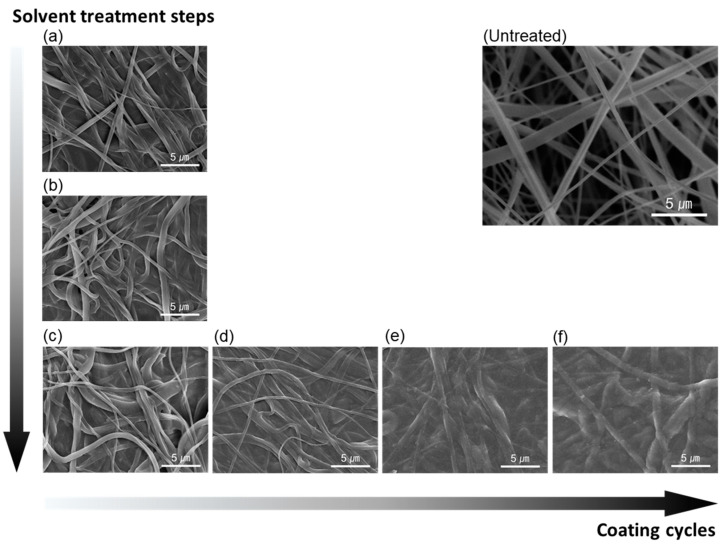
FE-SEM images of the untreated and treated samples (×5000); (**a**) P1, (**b**) PD1, (**c**) PD1-D, (**d**) PD2-D, (**e**) PD3-D, (**f**) PD4-D.

**Figure 8:**
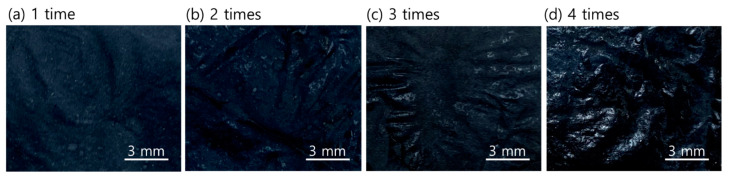
Actual images of the samples treated with PEDOT:PSS one to four times; (**a**) PD1-D, (**b**) PD2-D, (**c**) PD3-D, (**d**) PD4-D.

To investigate the microstructure change of the sample surface before and after treatment, FE-SEM images were analyzed (Figure 7). Observation of sample UT showed that the nanofibers were irregular and randomly collected. However, sample P1 and PD1 (Figure 7a,b, respectively) showed that the surface of the nanofibers was relatively more covered with PEDOT:PSS than UT. The nanofibers were still observed in sample PD1-D (Figure 7c), although PEDOT:PSS was treated on the surface of the sample. This is because the PSS particles were removed from the surface of the sample by DMSO treatment. Notably, the nanofibers of sample PD1-D, PD2-D, PD3-D, and PD4-D (Figure 7c–f, respectively) were less frequently observed as the number of coatings increased. In particular, the nanofibers were hardly observed in PD4-D, since the surface of the samples was completely covered by the PEDOT:PSS material. Figure 8 shows the actual appearance of sample PD1-D, PD2-D, PD3-D and PD4-D treated 1 to 4 times with PEDOT:PSS.

## 4. Conclusions

In this study, we aimed to fabricate a textile-based signal transmission line using PEDOT:PSS and nylon 6 nanofiber webs. The electrical and surface characteristics of the sample according to the treated conditions were investigated, and the optimum sample suitable for application as a signal transmission line was selected. For the selected samples, the signal waveforms were compared with the conventional copper wire in the time domain, and the characteristic curve was derived in the frequency domain. According to the obtained results, it was found that the electrical properties improved as the number of coatings of PEDOT:PSS increased, and the sample coated four times showed the lowest sheet resistance value. Secondly, the higher the DMSO treatment step, the sharper the decrease in sheet resistance. Accordingly, sample PD4-D showed the smallest sheet resistance (6.56 Ω/sq), which is much lower electrical resistance even compared to the non-oxidized graphene/PU nanofiber web (29.63 Ω/sq) [7], the PEDOT:PSS/PU nanofiber web (232.9~779.3 Ω/sq) [8], the PEDOT-coated polyamide fiber (approx. 2 kΩ) [27], and the glycerol/PEDOT:PSS-coated polyamide fiber (approx. 740 Ω) [28]. Based on the relationship with the thickness, it was confirmed that the improvement in electrical conductivity was caused by the increase in the amount of PEDOT:PSS deposition due to repeated coating, and the removal of the insulating PSS due to the increase in the solvent treatment step. Thirdly, for the performance of signal transmission, it was found that the signal waveforms of sample PD2-D, PD3-D, and PD4-D were almost identical to those of copper wires. In addition, their characteristic curves of frequency showed attenuation in the 3 GHz range, and it was confirmed that they can be used up to 15 MHz. Finally, as a result of the surface properties, it was confirmed that all samples were successfully coated with PEDOT:PSS and DMSO. Therefore, this study implies that it is possible to develop a textile-based signal transmission line for implementing a smart wearable system by using the PEDOT:PSS/Nylon 6 nanofiber web. As such, this study provides new options for controlling the electrical characteristics of nanofiber web-based signal transmission lines using ICPs. Further studies on improving high frequency characteristics, such as the placement of ground lines and interconnection methodology, are expected to add significance to this study for wearable technology.

## Figures and Tables

**Table 1:** Properties of the nylon 6 nanofiber web ^1^.

Substrates	Nylon 6 Nanofiber Web
Fiber diameter (nm)	200–500
Thickness (μm)	23
Weight (g/m^2^)	30
Melting point (°C)	220
Manufacturing technique	Centrifugal spinning

^1^ provided from the manufacturer (Paradam, Czech Republic).

**Table 2:** Specifications of the poly(3,4-ethylenedioxythiophene):poly(styrenesulfonate).

Characteristics	
Concentration (wt%)	1.3
Solid content (wt%)	1.2–1.4
PEDOT to PSS ratio	1:1.6
Conductivity (S/cm)	1
Density (g/cm^3^)	1
Form	Liquid
Color	Dark Blue

**Table 3:** Experimental design for the conductive textiles.

Sample	Coating Cycle	DMSO Treatment Steps	
		1-Step Treatment	2-Step Treatment
P1	1	-	-
PD1	1	0.5 mL, Adding	-
PD1-D	1	0.5 mL, Adding	20 mL, Dipping ^1^
PD2-D	2	0.5 mL, Adding	20 mL, Dipping ^1^
PD3-D	3	0.5 mL, Adding	20 mL, Dipping ^1^
PD4-D	4	0.5 mL, Adding	20 mL, Dipping ^1^

^1^ Dipping in DMSO bath.

**Table 4:** Linear regression equations of the samples.

Sample	R^2^	Equation
PD1, PD2, PD3, PD4	0.981	Y = −38.731X + 365.281 (*p* < 0.01)
PD1-D, PD2-D, PD3-D, PD4-D	0.931	Y = −7.137X + 56.133 (*p* < 0.05)

**Table 5:** Pearson’s correlation coefficient between I/O signals of the samples.

		Input Signal		
		PD2-D	PD3-D	PD4-D
Output signal	PD2-D	1.000 **		
	PD3-D		0.999 **	
	PD4-D			0.999 **

** *p* < 0.01.

**Table 6:** Pearson’s correlation coefficient between output signals of the sample and copper wire.

Sample	Copper Wire
PD2-D	0.999 **
PD3-D	0.999 **
PD4-D	0.999 **

** *p* < 0.01.

**Table 7:** Frequency of specimens corresponding to the −3 dB.

Specimen	Frequency (MHz)
PD2-D	14.73
PD3-D	15.09
PD4-D	14.74

## Data Availability

Data sharing not applicable. No new data were created or analyzed in this study. Data sharing is not applicable to this article. Page: 12.
